# Interface-Engineered Ni-Coated CdTe Heterojunction Photocathode for Enhanced Photoelectrochemical Hydrogen Evolution

**DOI:** 10.1021/acsami.3c01476

**Published:** 2023-04-20

**Authors:** Jing-Xin Jian, Luo-Han Xie, Asim Mumtaz, Tom Baines, Jonathan D. Major, Qing-Xiao Tong, Jianwu Sun

**Affiliations:** † Department of Physics, Chemistry and Biology (IFM), Linköping University, SE-58183 Linköping, Sweden; ‡ College of Chemistry and Chemical Engineering, Key Laboratory for Preparation and Application of Ordered Structural Material of Guangdong Province, and Guangdong Provincial Key Laboratory of Marine Disaster Prediction and Prevention, Shantou University, Shantou 515063, P. R. China; § School of Physics, Electronics & Technology, University of York, Heslington, York YO10 5DD, U.K.; ∥ Department of Physics, Stephenson Institute for Renewable Energy, University of Liverpool, Liverpool L69 7ZF, U.K.

**Keywords:** cadmium telluride, photoelectrochemical, water splitting, hydrogen evolution, interface engineering

## Abstract

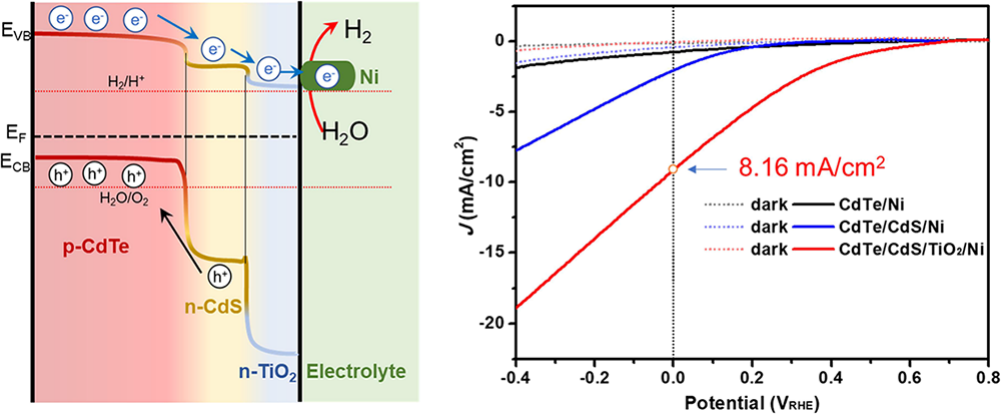

Photoelectrochemical (PEC) water splitting for hydrogen production using the CdTe photocathode has attracted much interest due to its excellent sunlight absorption property and energy band structure. This work presents a study of engineered interfacial energetics of CdTe photocathodes by deposition of CdS, TiO_2_, and Ni layers. A heterostructure CdTe/CdS/TiO_2_/Ni photocathode was fabricated by depositing a 100-nm *n*-type CdS layer on a *p*-type CdTe surface, with 50 nm TiO_2_ as a protective layer and a 10 nm Ni layer as a co-catalyst. The CdTe/CdS/TiO_2_/Ni photocathode exhibits a high photocurrent density (*J*_ph_) of 8.16 mA/cm^2^ at 0 V versus reversible hydrogen electrode (V_RHE_) and a positive-shifted onset potential (*E*_onset_) of 0.70 V_RHE_ for PEC hydrogen evolution under 100 mW/cm^2^ AM1.5G illumination. We further demonstrate that the CdTe/CdS *p*–*n* junction promotes the separation of photogenerated carriers, the TiO_2_ layer protects the electrode from corrosion, and the Ni catalyst improves the charge transfer across the electrode/electrolyte interface. This work provides new insights for designing noble metal-free photocathodes toward solar hydrogen development.

## 1 Introduction

Due to the high energy density of 142 MJ/kg and a clean exhaust (water), solar-driven photoelectrochemical (PEC) hydrogen (H_2_) production from water splitting has vast potential. Solar-driven PEC water splitting provides a sustainable and storable energy source assisting in reducing society’s dependence on fossil fuels, consequently reducing CO_2_ emissions and improving the ecological environment.^1−6^ The key part of the PEC water splitting cell is the semiconductor photoelectrode, which is required to efficiently absorb and convert solar energy into electrons and holes. Although much research effort has been dedicated to the investigation of different semiconductor photoelectrodes,^7−20^ it is still very challenging to develop a low-cost, robust, and highly efficient semiconductor photoelectrode to enable a practical solar hydrogen production.^21,22^

Cadmium telluride (CdTe) is an attractive candidate for hydrogen production via PEC water splitting due to its excellent optical absorption characteristics and band structure.^23−29^ As a direct semiconductor with a band gap of 1.5 eV, CdTe exhibits a wide range absorption from ultraviolet to infrared sunlight (until 830 nm) and a high absorption coefficient of >10^4^ cm^–1^ at the wavelengths smaller than 800 nm.^30,31^ It has been extensively demonstrated that CdTe is an excellent absorbing material in the application of photovoltaic devices.^32−35^ Since the pioneering work of Ohashi et al. in 1977,^36,37^*p*-type CdTe as a photocathode for the PEC water splitting has also attracted much interest. Recently, it has been demonstrated that the CdTe-based multilayer photocathodes with the deposited noble metal platinum (Pt) as the hydrogen evolution reaction (HER) co-catalyst exhibit an increased photocurrent and a significant shift of the onset potential.^30,38−42^ It is reported that the Au/Cu/CdTe (CdCl_2_)/CdS/Pt photocathode has shown a remarkably high incident photon-to-current conversion efficiency (IPCE) of >95% at 560–660 nm with an applied potential of 0 V versus reversible hydrogen electrode (V_RHE_).^30^ However, most reported CdTe-based photocathodes employed noble-metal Pt as the HER catalyst,^30,41,42^ which increases the cost of the PEC cells and hinders the future practical applications for low-cost hydrogen production. Moreover, the CdS layer generally suffers from corrosive degradation, and CdS/Pt overlayers were removed during the PEC tests, resulting in the decrease of the photocurrent and overall instability of the PEC device. Therefore, the most reported CdTe photocathodes employed a multilayered structure, which has shown the improvement of the overall PEC performance. However, it is still not clear how each layer plays the role in enhancing the PEC water reduction reaction.

In this work, we introduce a new strategy to fabricate a CdTe/CdS heterojunction photocathode and to enhance its PEC water splitting performance by depositing the earth-abundant and low-cost materials. Moreover, through engineering the overlayers of CdTe heterojunction photocathode, we investigate how each layer affects its overall PEC performance. Briefly, CdTe-based photocathodes were fabricated by depositing an *n*-type CdS layer on *p*-type CdTe forming a *p*–*n* heterojunction. The *n*-type TiO_2_ layer was deposited on either CdTe or CdS as a protective layer for comparison. Following, nickel (Ni), as the HER co-catalyst, was deposited on the CdTe-based photocathodes by vacuum evaporation. The benefit of using Ni is that it is earth-abundant and low-cost as compared to Pt. The optimal CdTe/CdS/TiO_2_/Ni photocathode exhibits a high photocurrent density (*J*_ph_) of 8.16 mA/cm^2^ at 0 V_RHE_ under 100 mW/cm^2^ AM1.5G illumination. Mechanism investigation revealed that the *p*–*n* junction of CdTe/CdS promotes the separation of photogenerated carriers, the TiO_2_ layer protects the photocathodes from corrosion, and the Ni co-catalyst improves the charge transfer across the electrode/electrolyte interface.

## 2 Experimental Section

### 2.1 Preparation of CdTe-Based Photocathodes

A number of different CdTe-based photocathodes were fabricated for this comparative study, including CdTe, CdTe/Ni, CdTe/CdS/Ni, CdTe/TiO_2_/Ni, and CdTe/CdS/TiO_2_/Ni. All photocathodes were deposited on NSG Ltd. soda lime TEC 15 glass (Florine-doped SnO_2_ (FTO)-coated glass). The CdTe layer was grown using close space sublimation (CSS), using a source and substrate temperature of 610 and 510 °C, respectively. The growth was performed with an initial higher pressure of 30 Torr N_2_ for 14 min, followed by a lower pressure growth at 1 Torr for 30 s. The purpose for this two-step process was to have larger grain sizes under higher pressure conditions and minimize pin holes in the film using lower pressure.^43^ The CdTe films were subsequently chlorine-treated to passivate the grain boundaries and have the effect of slight recrystallization of the CdTe grains, both of which improve their optoelectronic properties.^34^ For the chlorine treatment, MgCl_2_ was spray-deposited and subsequently annealed in an air ambient at 430 °C for 20 min.^33^ An etch step is then necessary to remove any surface contaminants. A wet etch using nitric–phosphoric acid (NP) solution is applied for 15 s.

The CdS layers were deposited via radio frequency (RF) magnetron sputtering with a target thickness of 80 nm. Sputtering took place at room temperature with a chamber pressure of 5 mTorr (0.66 Pa) using sputtering gas of Ar and a target power density of 1.32 W cm^–2^. A chamber base pressure of 1.9 × 10^–5^ Torr (2.53 mPa) was reached. The TiO_2_ layers were deposited using a spin coating technique over two stages. A solution of titanium isopropoxide in ethanol was used. The first step used a 0.15 M solution deposited using spin-coating at 3000 rpm^–1^ for 30 s and then annealed at 110 °C for 10 min. The next step was a deposition of a 0.3 M solution again using the same spin coating conditions, also followed by the same anneal step as before. The resultant layer was heated in air at 550 °C for a duration of 30 min. These steps followed a similar procedure as completed in previous studies.^35^ The target thickness for TiO_2_ was 50 nm although some variation due to surface roughness was anticipated.

The Ni nanoparticles were deposited on the surface of CdTe, CdTe/CdS, CdTe/TiO_2_, and CdTe/CdS/TiO_2_ photocathodes by vacuum evaporation. The thickness of the Ni layer was adjusted to optimize the PEC performance of Ni-coated CdTe/CdS/TiO_2_ photocathodes. The results show that CdTe/CdS/TiO_2_ coated with a 10-nm thick Ni layer exhibits the best PEC performance, as shown in Figure S1. SEM images were collected using a LEO 1550 Gemini instrument with an X-Max silicon drift detector (Oxford instruments).

### 2.2 PEC Characterizations

PEC measurements were carried out in a typical three-electrode system containing a counter electrode of Pt plate (1 × 1 cm^2^), a reference electrode of Ag/AgCl (saturated KCl), and a CdTe-based working electrode, using a potentiostat (Princeton Applied Research, VersaSTAT 3). The electrolyte of 0.1 M NaH_2_PO_4_ (NaPi, pH = 5.0) solution was degassed by bubbling argon (99.999%) for over 30 min. Standard simulated sunlight (AM1.5G, 100 mW/cm^2^) was supplied from a AAA solar simulator (LOT-Quantum Design GmbH). Current density-potential (*j*–*V*) measurements were carried out at a scan rate of 30 mV/s with chopped illumination. The measured potential with respect to Ag/AgCl (V_Ag/AgCl_) was converted to the potential versus reversible hydrogen electrode (V_RHE_) using the following equation: V_RHE_ = V_Ag/AgCl_ + 0.197 + 0.059 × pH. The evolved H_2_ gas was detected by a microgas chromatograph (Agilent Technologies 490 Micro GC) at 0 V_RHE_ under steady-state AM1.5G 100 mW/cm^2^ illumination. The Faradaic efficiency of H_2_ was determined by a comparison of the detected volume of H_2_ gas and the calculated volumes of H_2_ gas with a theoretical 100% faradaic efficiency.

## 3 Results and Discussion

### 3.1 PEC Performance of the Pristine CdTe Photocathode

**Figure 1:**
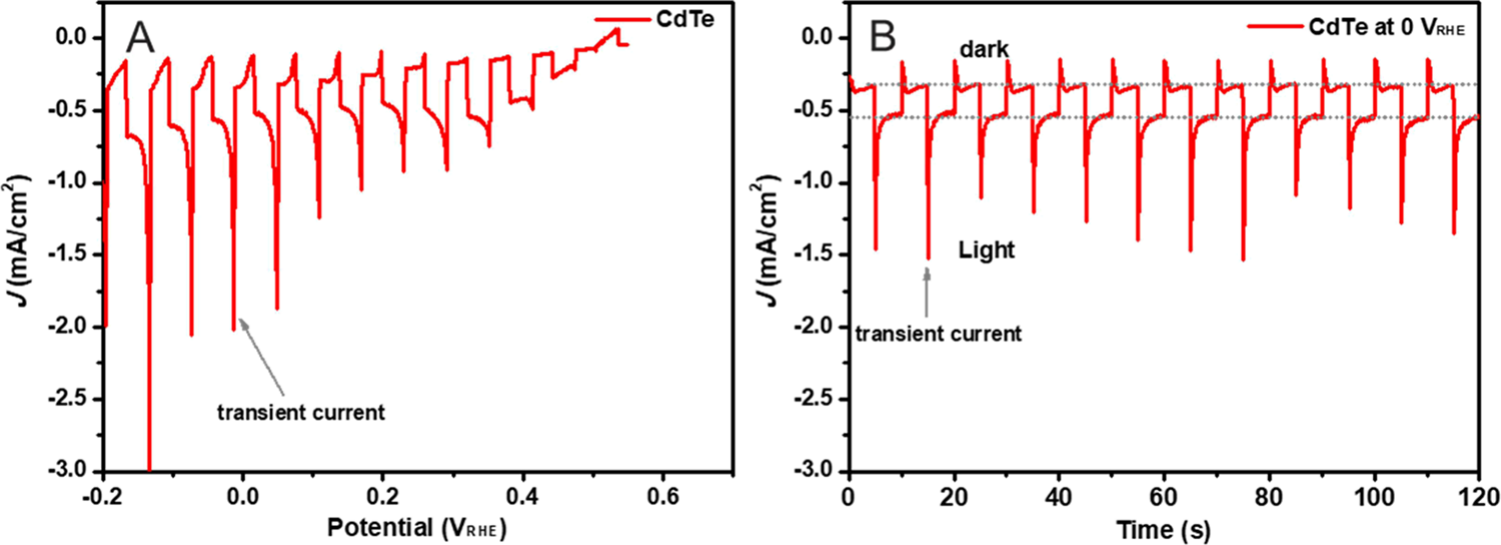
Current density–potential (*J*–*V*) curve (A) and current density–time (*J*–*t*) curve (B) of the pristine CdTe photocathode in 0.1 M NaPi electrolyte solution (pH = 5) under chopped AM1.5G 100 mW/cm^2^ illumination.

Figure 1 exhibits the PEC performance of the pristine CdTe photocathode in an Ar-purged 0.1 M NaH_2_PO_4_ (NaPi) electrolyte (pH = 5.0). Current density–potential (*J*–*V*) and current density–time (*J*–*t*) curves were measured under the chopped 100 mW/cm^2^ AM1.5G illumination so that the dark and photo-current could be monitored simultaneously. As shown in Figure 1, the pristine CdTe photocathode exhibits a significant transient photocurrent under chopped illumination, indicating a strong charge recombination and the sluggish HER catalytic performance at the surface of CdTe.^44,45^ Due to surface corrosion, the pristine CdTe photocathode exhibited a large dark current of ∼0.25 mA/cm^2^, which results in a rather small photocurrent density (*J*_ph_) of 0.25 mA/cm^2^ at 0 V_RHE_ (Figure 1B).

### 3.2 Ni-Coated CdTe Heterojunction Photocathode

**Figure 2:**
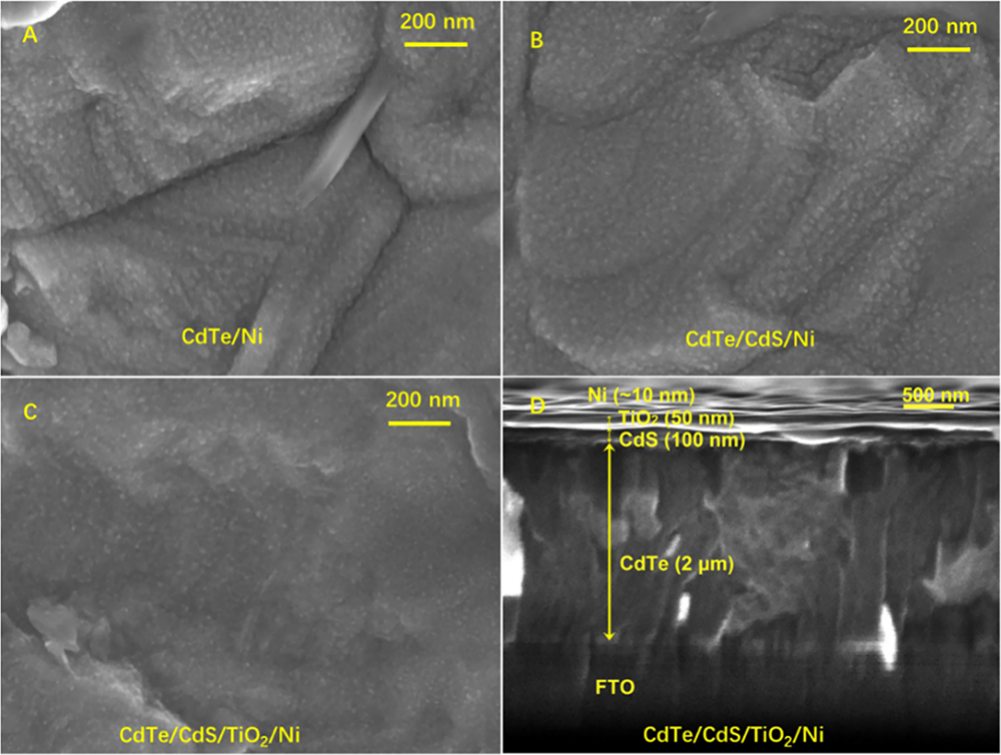
Top-view SEM images of the 10-nm Ni-coated CdTe (A), CdTe/CdS (B), and CdTe/CdS/TiO_2_ (C). (D) Cross-sectional view SEM image of the 10-nm Ni coated CdTe/CdS/TiO_2_.

To enhance the photocurrent of the CdTe photocathode, most reported works fabricated the CdTe/CdS heterojunction and used the noble metal Pt as the HER co-catalyst to improve H_2_ evolution efficiency.^30,39−42,46^ In this work, we employed the earth-abundant and low-cost metal Ni as the HER co-catalyst to boost the overall PEC performance of the CdTe-based photocathode. To optimize the thickness of the Ni layer, we deposited 2, 5, 10, and 50 nm Ni layers on the CdTe/CdS/TiO_2_ photocathodes, respectively. The chopped *J*–*V* curves shown in Figure S1 demonstrated that CdTe/CdS/TiO_2_ coated with the 10 nm thick Ni layer exhibits the best PEC performance (the highest photocurrent density and the largest on-set potential). To elucidate the role of each layer on the improvement of PEC performance, we deposited the 10 nm Ni layer on the surface of CdTe, CdTe/CdS, and CdTe/CdS/TiO_2_, respectively, for a comparative study. Figure 2A–C shows the surface morphologies of the 10 nm Ni-coated CdTe, CdTe/CdS, and CdTe/CdS/TiO_2_, indicating the identical Ni nanoparticles deposited on their surfaces. The thickness of each layer of the CdTe(2 μm)/CdS(100 nm)/TiO_2_(50 nm)/Ni(∼10 nm) photocathode is shown in the cross-sectional SEM image (Figure 2D).

**Figure 3:**
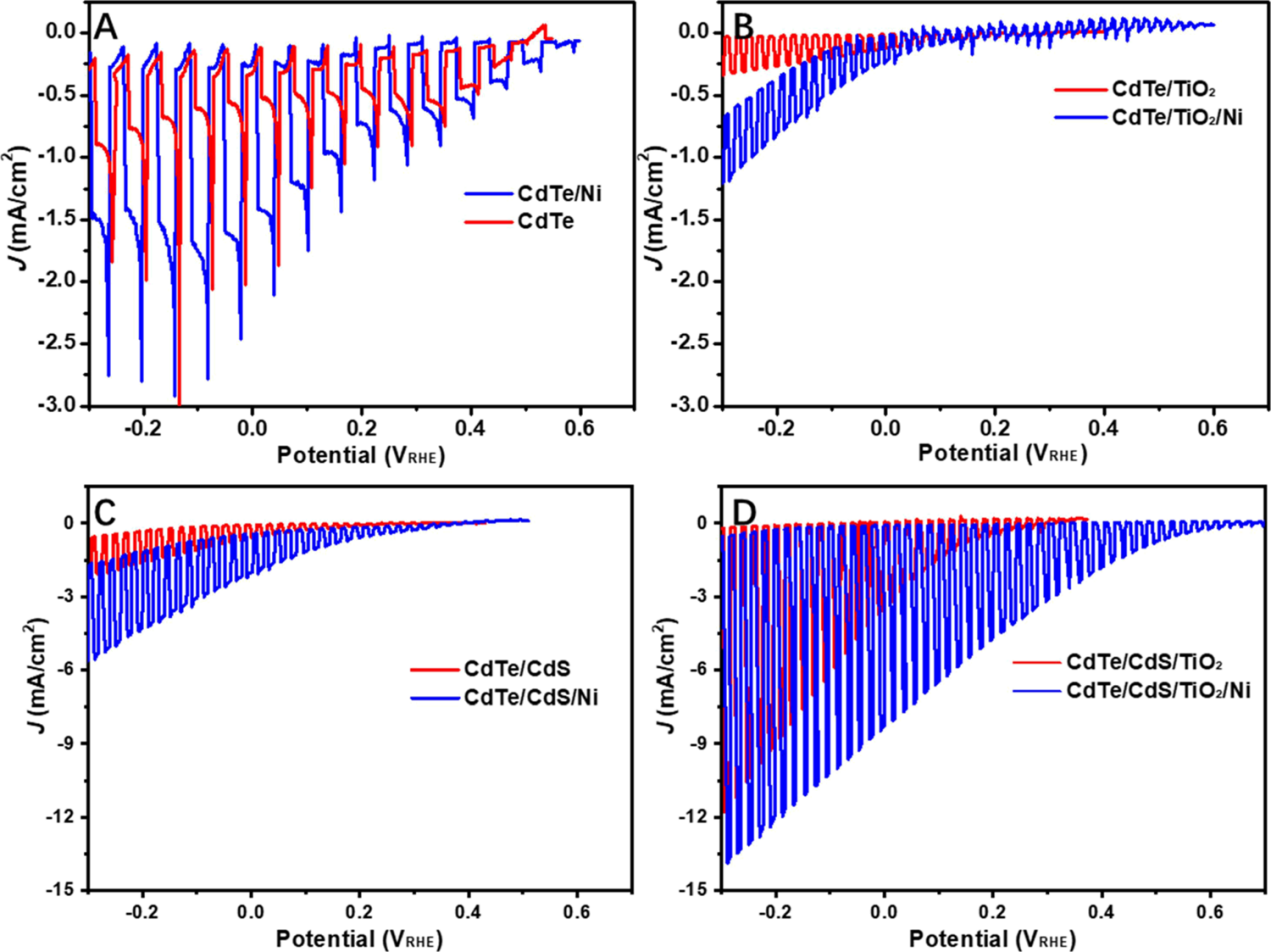
*J*–*V* curves of CdTe (A), CdTe/TiO_2_ (B), CdTe/CdS (C), and CdTe/CdS/TiO_2_(D) photocathodes with (blue lines) and without (red lines) 10 nm Ni co-catalysts, in 0.1 M NaPi electrolyte solution (pH = 5) and under chopped AM1.5G 100 mW/cm^2^ illumination.

Figure 3 compares the *J*–*V* curves of CdTe, CdTe/CdS, and CdTe/CdS/TiO_2_ photocathodes with and without the 10 nm Ni co-catalyst. As shown in Figure 3A, the CdTe/Ni photocathode exhibits a slightly positive-shifted onset potential (*E*_onset_) of 0.55 V_RHE_ and a high *J*_ph_ of 1.25 mA/cm^2^, which is five times higher than that of the pristine CdTe photocathode. The result indicates that the Ni co-catalyst promotes the H_2_ evolution activities of the CdTe photocathode. However, the CdTe/Ni photocathode still shows a significant transient current and the same *J*_dark_ with respect to the pristine CdTe photocathode. This suggests that charge recombination and surface corrosion issues still exist, which limits the improvement of photocurrent.

To suppress the charge recombination and corrosion during PEC water reduction, the interfaces of CdTe photocathodes were engineered by introducing an *n*-type TiO_2_ layer with/without an *n*-type CdS layer on top of CdTe.^40,47−50^ The photocurrents of these heterojunction photocathodes were measured at the same conditions as the CdTe photocathode. As seen in Figure 3B, the TiO_2_-coated CdTe (CdTe/TiO_2_) photocathode exhibits an extraordinarily low *J*_dark_ of 0.00 mA/cm^2^ at 0 V_RHE_, a very low *J*_ph_ of 0.13 mA/cm^2^ at 0 V_RHE_ and a small *E*_onset_ of 0.38 V_RHE_. In contrast, the CdS-coated CdTe (CdTe/CdS) photocathode exhibits a low *J*_dark_ of 0.06 mA/cm^2^ at 0 V_RHE_, an improved *J*_ph_ of 0.53 mA/cm^2^ at 0 V_RHE_, and *E*_onset_ of 0.45 V_RHE_ (Figure 3C). It should be noted that the transient current behaviors of CdTe/TiO_2_ and CdTe/CdS photocathodes are significantly suppressed under chopped illumination due to the enhanced charge separation by the built-in electric field in the formed *p*–*n* heterojunction. Figure 3 shows that CdTe/CdS exhibited higher photocurrent than CdTe/TiO_2_.

The 10 nm thick Ni catalyst layers were deposited on the CdTe/TiO_2_ and CdTe/CdS photocathodes to improve their HER activities. In the *J*–*V* curves under chopped illumination, the CdTe/CdS/Ni photocathode shows a *J*_dark_ of 0.43 mA/cm^2^ at 0 V_RHE_, an enhanced *J*_ph_ of 1.56 mA/cm^2^ at 0 V_RHE_, and *E*_onset_ of 0.50 V_RHE_ (Figure 3C), while the CdTe/TiO_2_/Ni photocathode shows a low *J*_dark_ of 0.02 mA/cm^2^ at 0 V_RHE_, *J*_ph_ of 0.22 mA/cm^2^ at 0 V_RHE_, and *E*_onset_ of 0.44 V_RHE_ (Figure 3B), respectively. The Ni-coated photocathodes exhibit enhanced photocurrent than its counterpart without the Ni catalyst, indicating that the Ni catalyst is a promising HER catalyst to boost H_2_ evolution. The increased dark current of Ni-coated photocathodes was due to surface corrosion.

### 3.3 CdTe/CdS/TiO_2_/Ni Photocathode

**Figure 4:**
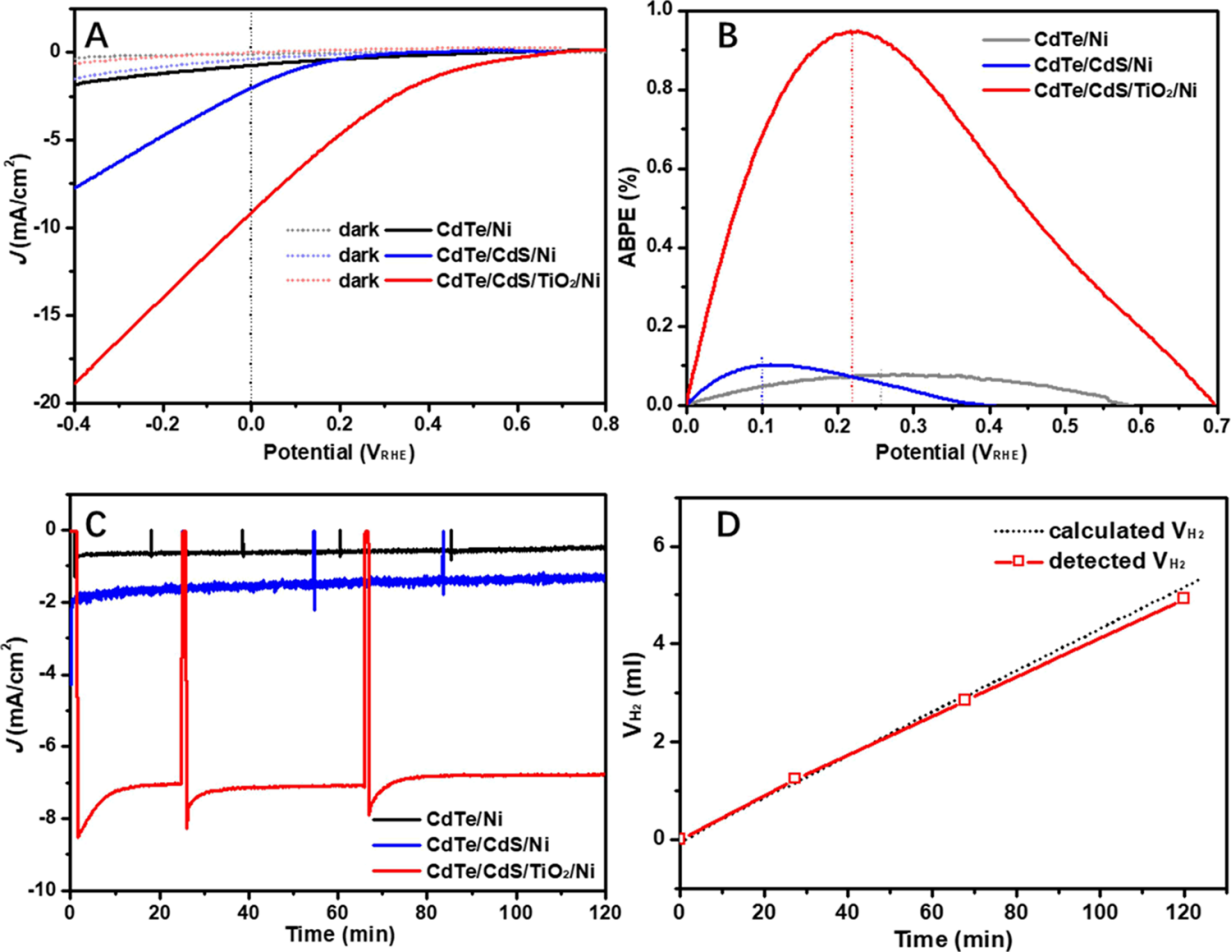
*J*–*V* curves (A) and applied bias photo-to-current efficiency (ABPE) curves (B) of CdTe/Ni, CdTe/CdS/Ni, and CdTe/CdS/TiO_2_/Ni photocathodes in 0.1 M NaPi electrolyte solution (pH = 5) under steady state AM1.5G 100 mW/cm^2^ illumination.(C) *J*–*t* curve of CdTe/Ni, CdTe/CdS/Ni, and CdTe/CdS/TiO_2_/Ni photocathodes in 0.1 M NaPi electrolyte solution (pH = 5) under stated AM1.5G 100 mW/cm^2^ illumination. (D) Measured H_2_ volume of the CdTe/CdS/TiO_2_/Ni photocathode under the condition of (C). The black dotted line shows the theoretical volume of H_2_ with 100% faradaic efficiencies.

Although the CdTe/CdS *p*–*n* heterojunction improves the photoelectrochemical water splitting performance, it still suffers from poor stability. To address this issue, a CdTe/CdS/TiO_2_/Ni photocathode was fabricated to synergistically enhance both the photocurrent and stability. As shown in Figure 3D, the CdTe/CdS/TiO_2_ photocathode exhibits a significantly enhanced *J*_ph_ of 3.68 mA/cm^2^ at 0 V_RHE_ and a negligibly low dark current. With deposited 10 nm Ni, the CdTe/CdS/TiO_2_/Ni photocathode exhibits the negligibly low dark current, the largest *E*_onset_ of 0.70 V_RHE_, and the highest *J*_ph_ of 8.16 mA/cm^2^ at 0 V_RHE_ under 100 mW/cm^2^ AM1.5G illumination among all photocathodes (Table 1). This photocurrent is 2.22-folds of the CdTe/CdS/TiO_2_ photocathode and 32.6-folds of the CdTe photocathode. The photocurrent in this Ni-coated photocathode is comparable with the reported results of the noble metal Pt-coated CdTe photocathode (Table S1).^39^Figure 4A compares the *J*–*V* curves of CdTe/Ni, CdTe/CdS/Ni, and CdTe/CdS/TiO_2_/Ni photocathodes under steady state AM1.5G 100 mW/cm^2^ illumination, which gives the same photocurrent density *J*_ph_ and *E*_onset_ as obtained under chopped illumination (Figure 3). These results indicate that the CdTe/CdS *p*–*n* heterojunction distinctly outperforms CdTe/TiO_2_ to achieve a higher photocurrent, the TiO_2_ layer forms a protective layer that significantly reduces the dark current from corrosion, and the Ni co-catalyst significantly enhances the HER activity for hydrogen generation.

**Table 1:** PEC Performance of the CdTe-Based Photocathodes

photocathodes	*A* (cm^2^)	*J*_dark_@0 V_RHE_ (mA/cm^2^)	*J*_ph_@0 V_RHE_ (mA/cm^2^)	*E*_onset_ (V_RHE_)	ABPE (%)	V_ABPEmax_ (V_RHE_)
CdTe	0.5	0.05	0.25	0.45	0.01	0.23
CdTe/Ni	0.5	0.06	1.25	0.45	0.08	0.26
CdTe/CdS	0.8	0.06	0.53	0.43	0.03	0.12
CdTe/CdS/Ni	1.0	0.43	1.56	0.50	0.10	0.10
CdTe/TiO_2_	0.9	0.00	0.13	0.38	0.01	0.12
CdTe/TiO_2_/Ni	0.5	0.02	0.22	0.44	0.02	0.15
CdTe/CdS/TiO_2_	0.9	0.04	3.68	0.45	0.18	0.11
**CdTe/CdS/TiO_2_/Ni**	**0.9**	**0.08**	**8.16**	**0.70**	**0.95**	**0.22**

Figure 4B shows the applied bias photo-to-current efficiency (ABPE) curves of CdTe/Ni, CdTe/CdS/Ni, and CdTe/CdS/TiO_2_/Ni photocathodes. ABPE is given by the following equation: ABPE = *J*_ph_ × V_RHE_/P_AM1.5G_, where P_AM1.5G_ is the incident illumination power density (AM1.5G 100 mW/cm^2^).^44^ The Ni-coated photocathodes demonstrated significantly higher ABPE than their counterparts without the Ni catalyst (Table 1). The optimal CdTe/CdS/TiO_2_/Ni photocathode exhibited a maximum ABPE of 0.95% at an applied potential of 0.22 V_RHE_, which is 5.28-folds of CdTe/CdS/TiO_2_ photocathode and 95-folds of CdTe photocathode, respectively.

The evolved H_2_ gas for the CdTe/CdS/TiO_2_/Ni photocathode was measured in a two-compartment cell. Under AM1.5G 100 mW/cm^2^ illumination, the chronoamperometry (*J*–*t*) curve of the CdTe/CdS/TiO_2_/Ni photocathode exhibits a reproducible current of around 8.16 mA/cm^2^ at 0 V_RHE_ (Figure 4C). The *J*–*t* curve confirms that the CdTe/CdS/TiO_2_/Ni photocathode was stable after over 120 min of illumination. Meanwhile, the amount of H_2_ gases was measured by gas chromatography to evaluate the faradaic efficiency of H_2_. As shown in Figure 4D, the CdTe/CdS/TiO_2_/Ni photocathode exhibits a ∼100% faradaic efficiency for H_2_ evolution. After the CdTe/CdS/TiO_2_/Ni photocathode has been stored for over one year, the *J–t* curve measurement for 14 h showed a repeatable and stable *J*_ph_ of around 6.9 mA/cm^2^ at 0 V_RHE_ under AM 1.5G illumination (Figure S2).

### 3.4 Understanding of the PEC Improvements of the Ni-Coated Heterojunction Photocathode

**Figure 5:**
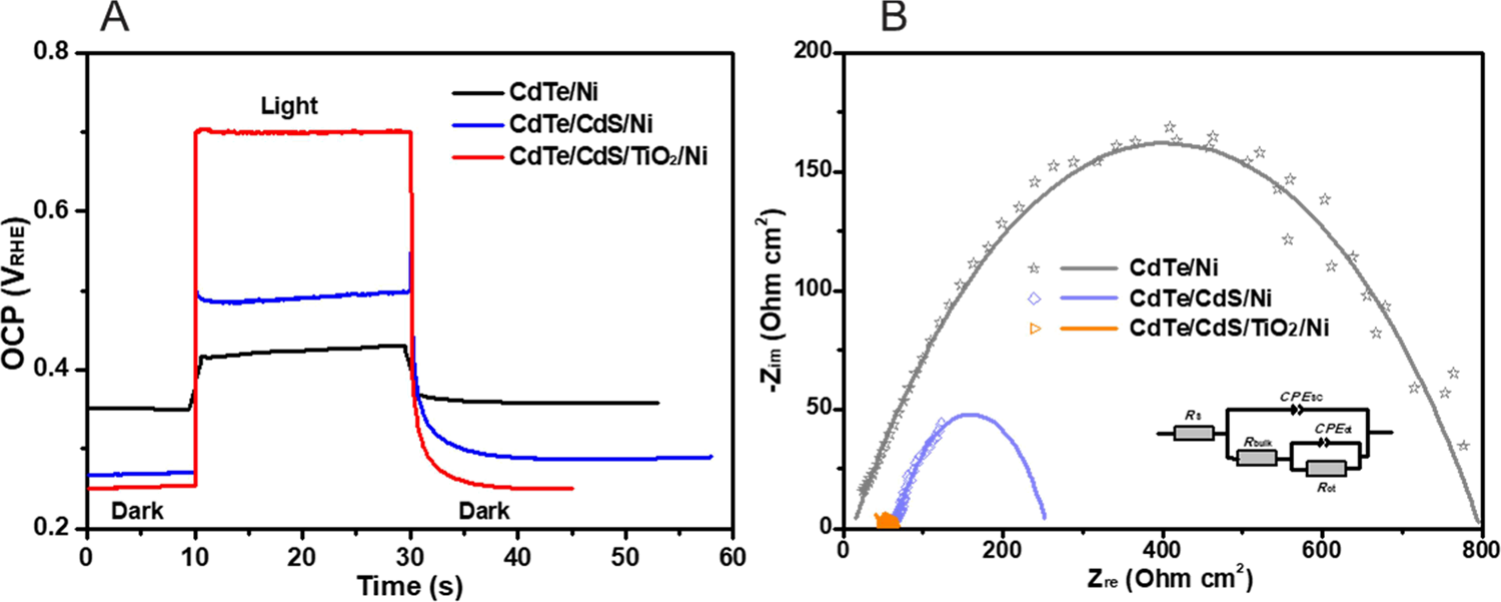
OCP (A) and Nyquist plots (B) of CdTe-based photocathodes with the Ni catalyst at 0 V_RHE_ under AM1.5G 100 mW/cm^2^ illumination.

To understand the enhanced PEC performance of Ni-coated CdTe heterojunction photocathodes, we performed photovoltage measurements under open-circuit potential (OCP) conditions (Figures 5 and S3). Under illumination, the photogenerated electron–hole pairs are separated by the built-in electric field in the space charge region. The electric field drives the majority carriers (holes) into the bulk of the semiconductor and the minority carriers (electrons) toward the photocathode/electrolyte interface, resulting in a photovoltage. As shown in Figures 5A and S3, all the CdTe-based photocathodes exhibit positive-shifted OCPs under illumination, which is a characteristic of the *p*-type CdTe photocathodes. The OCP measured in the dark and under illumination represents a photovoltage (*V*_ph_) generated in the photocathode. As summarized in Table 2, the *p*–*n* heterojunction CdTe/CdS and CdTe/TiO_2_ shows higher *V*_ph_ than the pristine CdTe, indicating that the *p*–*n* junction improves the separation of the photogenerated carriers. Moreover, the optimal CdTe/CdS/TiO_2_/Ni photocathode exhibits the highest *V*_ph_ of 0.45 V among the CdTe-based photocathodes, which is consistent with its enhanced photocurrent and positive-shifted onset potential. This is further confirmed by the incident photon-to-current efficiency (IPCE) measurements. As shown in Figure S4, the CdTe/CdS/TiO_2_/Ni photocathode exhibits a high IPCE of ∼65% in the range of 400–600 nm, which is significantly improved compared to the CdTe/CdS/TiO_2_ photocathode (IPCE: ∼40% in the range of 400–600 nm). With a synergetic effect of the *p*–*n* heterojunction and the Ni co-catalyst, the CdTe/CdS/TiO_2_/Ni photocathode promotes charge separation, and thus, more electrons can be swept to the photocathode/electrolyte interface to contribute to the photoelectrochemical H_2_ evolution.

**Table 2:** OCP and EIS Results of the CdTe-Based Photocathodes

photocathodes	*J*_ph_@0 V_RHE_ (mA/cm^2^)	*V*_ph_ (V)	*R*_tot_ (Ω*cm^2^)
CdTe	0.25	0.06	3990.9
CdTe/Ni	1.25	0.07	846.6
CdTe/CdS	0.53	0.11	848.2
CdTe/CdS/Ni	1.56	0.08	256.1
CdTe/TiO_2_	0.13	0.17	3721.6
CdTe/TiO_2_/Ni	0.22	0.15	2031.4
CdTe/CdS/TiO_2_	3.68	0.18	134.0
**CdTe/CdS/TiO_2_/Ni**	**8.16**	**0.45**	**44.9**

Electrochemical impedance spectroscopy (EIS) measurements were employed to study the charge-transfer properties at the photocathode/electrolyte interface. Figures 5B and S5 show the Nyquist plots of CdTe-based photocathodes at 0 V_RHE_ in the frequency range of 1–10^5^ Hz under AM1.5G 100 mW/cm^2^ illumination. The EIS data are fitted using the equivalent circuits shown in the inset of Figure 5B. The constant phase elements (*CPE*) are used instead of the standard capacitance (*C*) in equivalent circuits due to their non-ideal capacitive behavior in Bode phase plots with phase angles below 90^o^. To interpret the EIS data, the series resistance (*R*_s_), the charge-transfer resistance (*R*_bulk_) from the semiconductor bulk to its surface, the corresponding constant phase element of the space-charge capacitance (*CPE*_SC_), the charge-transfer resistance from the photocathode to the electrolyte (*R*_ct_), and its corresponding capacitance (*CPE*_ct_) are included in the equivalent circuit. A total resistance (*R*_tot_) is a sum of *R*_s_, *R*_bulk_, and *R*_ct_ (Tables 2 and S2). As depicted in Figure S5, the CdTe/CdS and CdTe/CdS/Ni photocathodes exhibit smaller semicircles (lower *R*_bulk_ and *R*_ct_) than the CdTe/TiO_2_ and CdTe/TiO_2_/Ni photocathodes, respectively, indicating that the CdTe/CdS/Ni heterostructure is beneficial for charge transfer toward the electrolyte.^51^ Among all photocathodes, the CdTe/CdS/TiO_2_/Ni exhibits the smallest *R*_tot_ (44.9 Ω*cm^2^), owing to the efficient charge separation by the *p*–*n* junction and the boosted electron transfer across electrode/electrolyte interface through the Ni catalyst. This clearly demonstrates that the heterojunction structure and Ni catalyst have a synergetic enhancement of the PEC water splitting performance of the CdTe photocathode.

**Figure 6:**
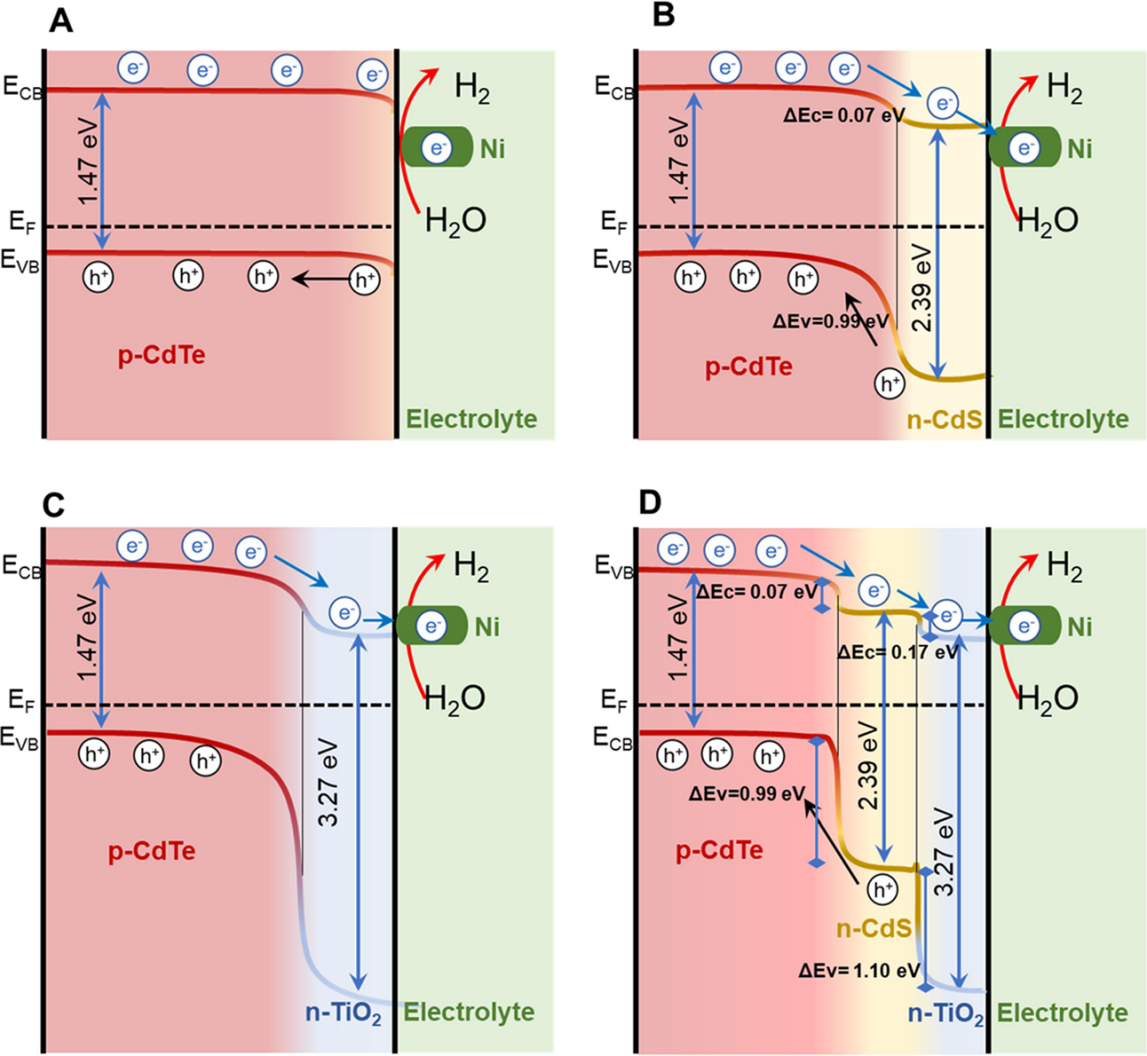
Band structure illustrations of the CdTe/Ni (A), CdTe/CdS/Ni (B), CdTe/TiO_2_/Ni(C), and CdTe/CdS/TiO_2_/Ni (D) photocathodes. *E*_F_: Fermi level (EF), CB: conduction band, VB: valence band.

To illustrate the effect of the *p*–*n* heterojunction on the improvement of PEC performance, the schematic energy band structures of the Ni-coated CdTe photocathodes are compared in Figure 6. According to the absorption measurements (Figure S6), the band gaps of CdTe, CdS, and TiO_2_ layers are determined to be ∼1.47, ∼2.39, and ∼3.27 eV, respectively. These optical band gaps are consistent with the reported values.^42^ The conduction band offset (Δ*E*_c_) and valence band offset (Δ*E*_V_) of the CdTe/CdS heterojunction were determined to be 0.07 eV and 0.99 eV, respectively, and Δ*E*_c_ = 0.17 eV and Δ*E*_V_ = 1.1 eV for the CdS/TiO_2_ heterojunction.^42^ As shown in Figure 6, the band bending at the CdTe/electrolyte interface forms a Schottky junction, which is the driving force for PEC water splitting. The formation of the *p*–*n* heterojunction results in the alignment of Fermi levels, which causes band bending where the built-in electric field promotes the charge separation and transport, thus enhancing the photovoltage and the overall PEC performance (Table 2). According to the EIS, IPCE, and photovoltage results, it is evident that the Ni co-catalyst significantly boosts the electron transfer toward the electrolyte for hydrogen generation (Figure 6B–D).

### 3.5 Stability of the Ni-Coated Heterojunction Photocathode

**Figure 7:**
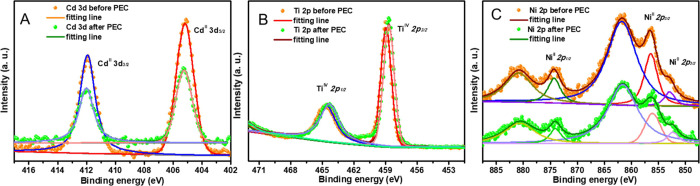
XPS spectra of Cd 3d (A), Ti 2p (B), and Ni 2p(C) of the CdTe/CdS/TiO_2_/Ni photocathode before and after the PEC measurement for 2-hour chronoamperometry at 0 V_RHE_ in 0.1 M NaPi electrolyte solution (pH = 5) under stated AM1.5G 100 mW/cm^2^ illumination.

To investigate the stability of the Ni-coated CdTe photocathode, the surface morphology and the surface composition and chemical states of the CdTe/CdS/TiO_2_/Ni photocathode were measured by SEM and X-ray photoelectron spectroscopy (XPS) before and after PEC measurements. The top view SEM images of CdTe/CdS/TiO_2_/Ni before and after PEC measurements show an unchanged surface morphology (Figure S7A,B). As shown in the XPS survey spectra (Figure S7C), the CdTe/CdS/TiO_2_/Ni photocathode exhibits identical peaks before and after PEC measurements, which are assigned to the Cd, Ti, Ni, Te, and S elements.^36^ The Cd 3d spectra shown in Figure 7A show two peaks at 405.2 and 411.9 eV, which have been attributed to the spin-orbit doublets of 3d_5/2_ and 3d_3/2_, respectively. The Ti 2p spectra in Figure 7B displays two spin-orbit doublets of Ti 2p_3/2_ (458.7 eV) and Ni 2p_1/2_ (464.4 eV). As shown in Figure 7C, the Ni 2p spectra display several fitting peaks, which are the typical characteristics of the presence of Ni^0^ (852.2 eV) and Ni^II^ with peaks of 2p_3/2_ (856.5 eV), Ni^II^ 2p_1/2_ (874.3 eV), and their satellites peaks (861.7 and 880.7 eV).^52^ The XPS spectra of the CdTe/CdS/TiO_2_/Ni photocathode clearly demonstrated that the Cd, Ti, and Ni elements exhibit identical peaks before and after PEC measurements. These results confirm a high stability of the CdTe/CdS/TiO_2_/Ni photocathode for PEC water splitting.

## 4 Conclusions

In conclusion, a Ni-coated heterostructure photocathode of CdTe/CdS/TiO_2_/Ni was fabricated by depositing a 100 nm *n*-type CdS layer on the *p*-type CdTe surface forming a *p*–*n* junction structure, followed by the deposition of 50 nm TiO_2_ as the protective layer and 10 nm Ni as the hydrogen-evolution co-catalyst. The prepared CdTe/CdS/TiO_2_/Ni photocathode exhibits a high *J*_ph_ of 8.16 mA/cm^2^ at 0 V_RHE_ and a positive-shifted *E*_onset_ of 0.70 V_RHE_ for photoelectrochemical hydrogen evolution under one simulated sunlight. OCP, EIS, and PEC stability studies indicated that the *p*–*n* junction of CdTe/CdS promotes the separation of photogenerated carriers, the TiO_2_ layer protects the electrode from corrosion, and the Ni catalyst improves the charge transfer across the electrode/electrolyte interface. This work provides new insights for designing noble-metal free photocathodes toward solar hydrogen development.

## Supporting Information Available

Optimization of Ni layer thickness, open-circuit potential, IPCE, electrochemical impedance spectroscopy results and analysis, absorption and Tauc plot, and XPS survey spectra (PDF)
