# Supplementary material for: Interface-Engineered Ni-Coated CdTe Heterojunction Photocathode for Enhanced Photoelectrochemical Hydrogen Evolution
Source: ACS Appl Mater Interfaces. 2023 Apr 20;15(17):21057–65. doi: 10.1021/acsami.3c01476 (PMC10165602; doi:10.1021/acsami.3c01476)
Supplement: Supplementary file 1 — am3c01476_si_001.pdf [file am3c01476_si_001.pdf]

Supporting Information

Interface Engineered Ni-coated CdTe Heterojunction Photocathode for Enhanced Photoelectrochemical Hydrogen Evolution

Jing-Xin Jian,[a,b] *Luo-Han Xie,*[b] *Asim Mumtaz,*[c] *Tom Baines,*[d] *Jonathan D Major,*[d] *Qing-Xiao Tong*[b] *and Jianwu Sun* [a,*]

[a] Department of Physics, Chemistry and Biology (IFM), Linköping University, SE-58183, Linköping, Sweden.

[b] College of Chemistry and Chemical Engineering, Key Laboratory for Preparation and Application of Ordered Structural Material of Guangdong Province, and Guangdong Provincial Key Laboratory of Marine Disaster Prediction and Prevention, Shantou University, Shantou, 515063, P. R. China.

[c] School of Physics, Electronics & Technology, University of York, Heslington, York, YO10 5DD, United Kingdom

[d] Department of Physics, Stephenson Institute for Renewable Energy, University of Liverpool, L69 7ZF, United Kingdom.

[*] Corresponding author: Jianwu Sun, e-mail: jianwu.sun@liu.se

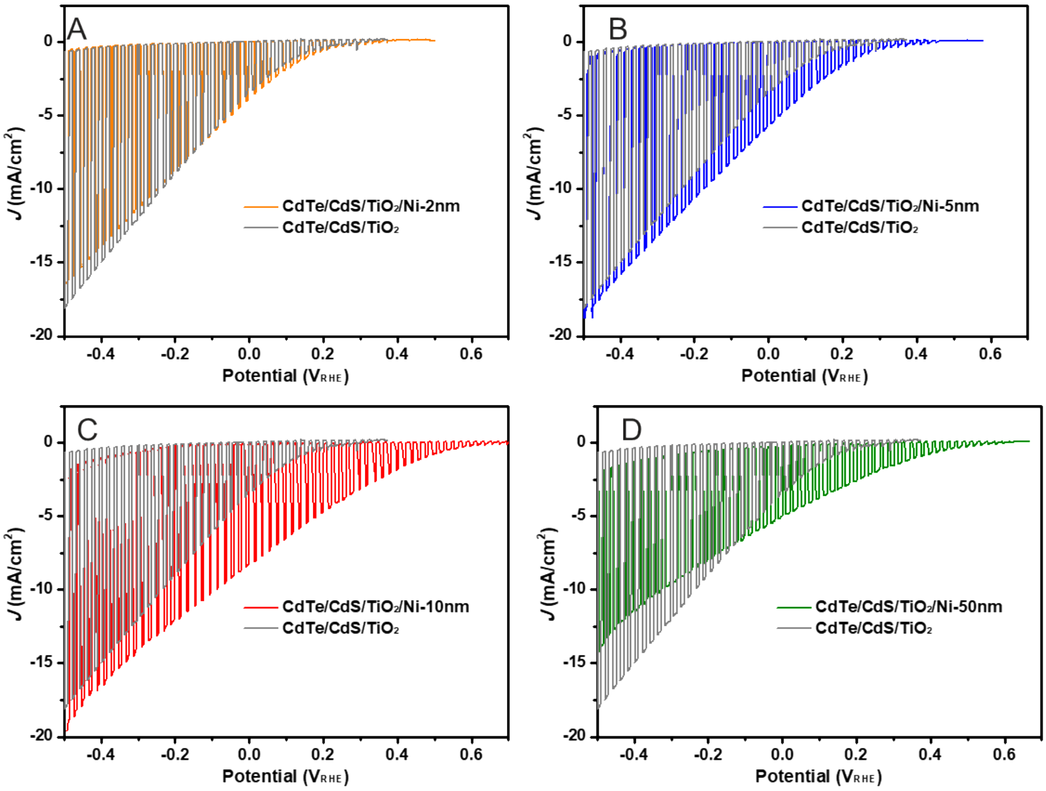

Figure S1. *J-V* curves of CdTe/CdS/TiO_2/Ni photocathodes with different thicknesses of the Ni layer (2, 5, 10 and 50 nm).

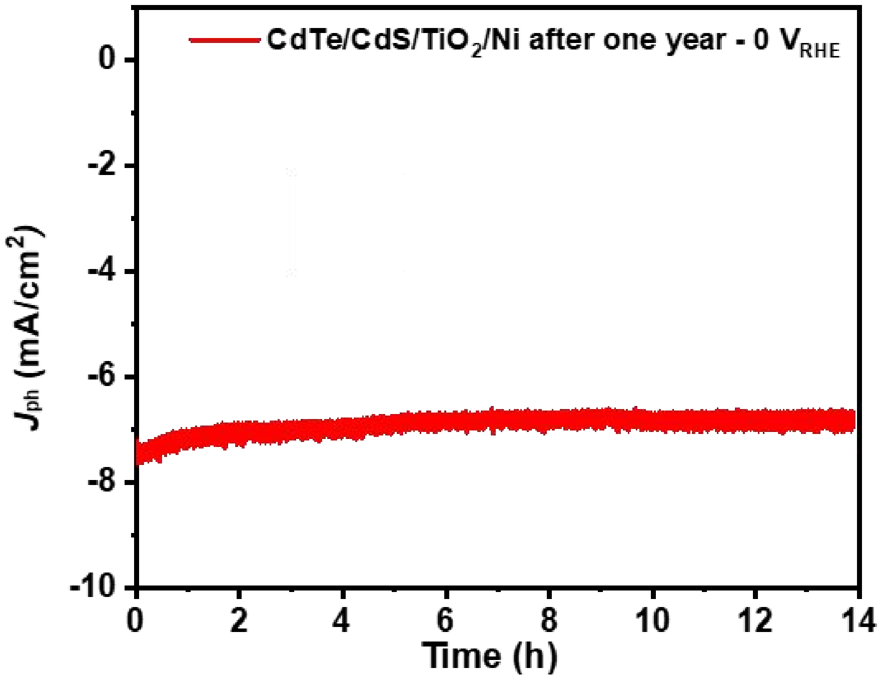

Figure S2. *J-t* curve of the $CdTe/CdS/TiO_2/Ni$ photocathode measured after one year, in 0.1 M NaPi electrolyte solution (pH = 5) under 100 mW/cm^2 AM 1.5G illumination.

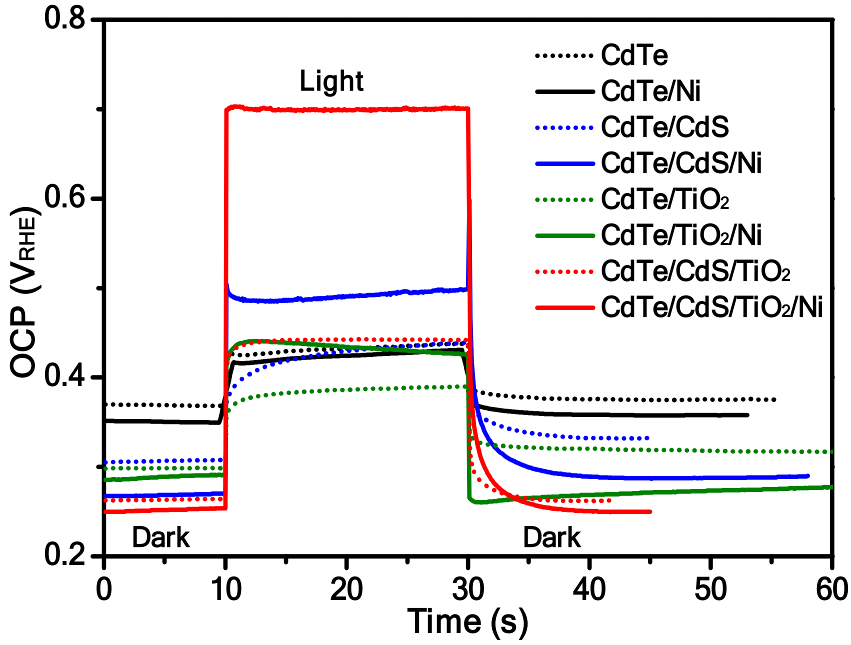

Figure S3. OCP of CdTe-based photocathodes at 0 V_{RHE} under AM1.5G, 100 mW/cm^2 illumination.

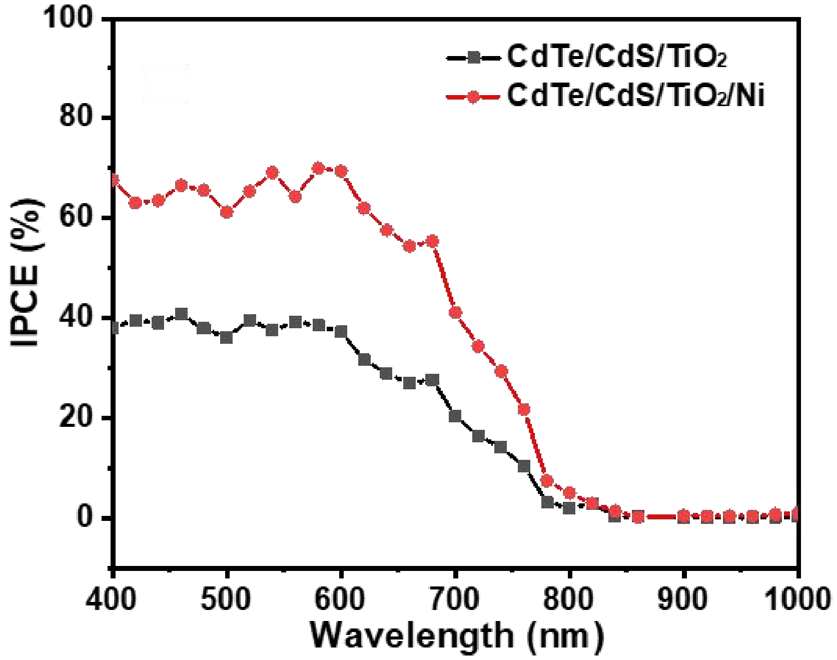

Figure S4. IPCE of $CdTe/CdS/TiO_2$ and $CdTe/CdS/TiO_2/Ni$ photocathodes measured at 0 V_{RHE} under monochromatic light (1.0 mW cm^{-2}) illumination from the Xe lamp equipped with a monochromator.

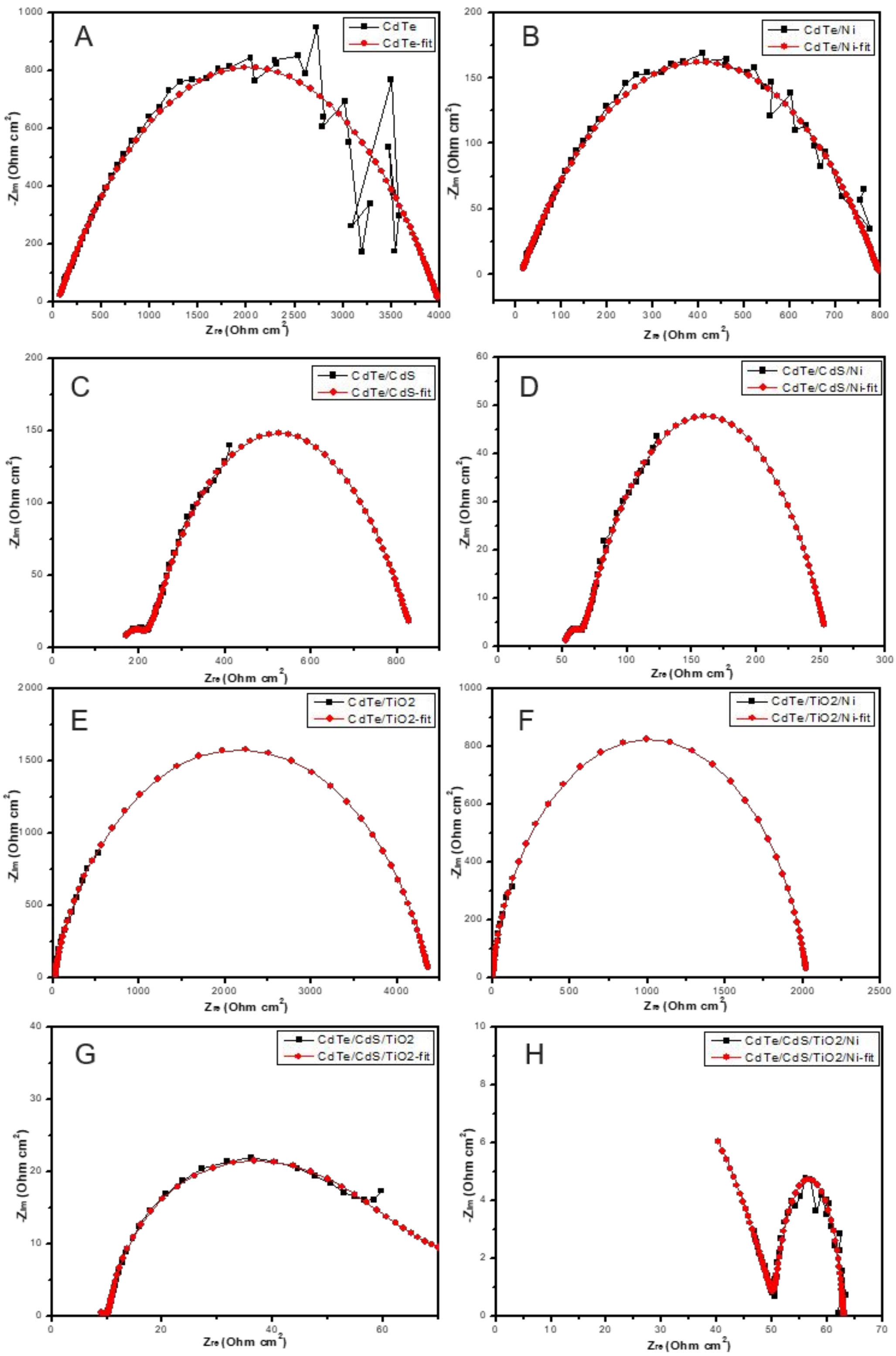

Figure S5. Nyquist plots of CdTe (A), CdTe/Ni (B), CdTe/CdS (C), CdTe/CdS/Ni (D), CdTe/TiO_2 (E), CdTe/TiO_2/Ni (F), CdTe/CdS/TiO_2 (G), and CdTe/CdS/TiO_2/Ni (H) photocathodes in the frequency range of 1–10^5 Hz under AM1.5G 100 mW/cm^2 illumination at 0 V_{RHE}.

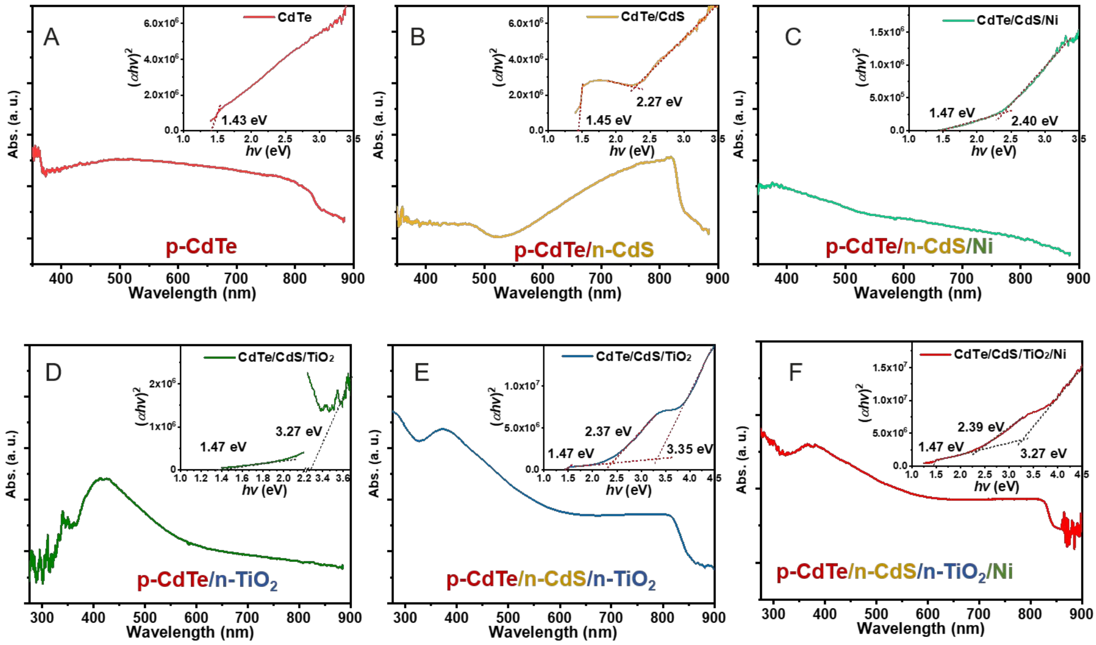

Figure S6. Absorption spectra and Tauc plot of CdTe (A), CdTe/CdS (B), CdTe/CdS/Ni (C), CdTe/TiO_2 (D), CdTe/CdS/TiO_2 (E) and CdTe/CdS/TiO_2/Ni (F).

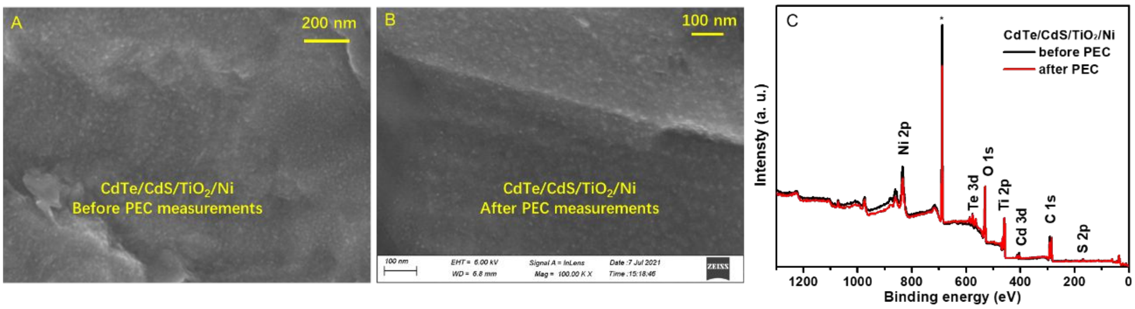

Figure S7. Top view SEM image of the CdTe/CdS/TiO_2/Ni photocathodes before (A) and after (B) PEC measurements. XPS survey spectra of the CdTe/CdS/TiO_2/Ni photocathode before and after PEC measurements (C). The star peak at 690 eV is the CF_2 from the plastic substrate.

Table S1. PEC performance of recently reported CdTe-based photocathodes.

Entries	J_{ph}@0V_{RHE} (mA/cm^2)	E_{onset}	ABPE (%)	**Reference**
CdTe/CdS/Pt	6.0	0.6 V_{RHE}	0.90%	39
Au/Cu/CdTe($CdCl_2$)/CdS/Pt	22.0	0.6 V_{RHE}	3.7%	30
CdTe/CdS/Pt	0.24	0.3 $V_{Ag/AgCl}$	--	41
CdTe/CdS/TiO_2/Pt	4.9	0.29 V_{RHE}	--	42
**CdTe/CdS/TiO_2/Ni**	**8.16**	**0.70** V_{RHE}	**0.95%**	**This work**

Table S2. EIS data of the CdTe-based photocathodes.

Photocathodes	R_s (Ω*cm^2)	R_{bulk} (Ω*cm^2)	R_{ct} (Ω*cm^2)	CPE_{ct}-T	CPE_{ct}-P	CPE_{sc}-T	CPE_{sc}-P	R_{tot} (Ω*cm^2)
**CdTe**	58.9	--	3932.0	--	--	1.14×10^{-5}	0.50	3990.9
**CdTe/Ni**	31.2	--	815.4	--	--	4.14×10^{-5}	0.52	846.6
**CdTe/CdS**	81.7	153.3	613.2	8.07×10^{-4}	0.60	1.72×10^{-4}	0.35	848.2
**CdTe/CdS/Ni**	51.4	15.8	188.9	2.86×10^{-3}	0.60	2.31×10^{-4}	0.50	256.1
**CdTe/TiO_2**	22.0	18.6	3681.0	7.17×10^{-5}	0.91	1.19×10^{-4}	0.78	3721.6
**CdTe/TiO_2/Ni**	3.4	1.1	2026.9	3.61×10^{-4}	0.93	1.95×10^{-4}	0.75	2031.4
**CdTe/CdS/TiO_2**	3.7	7.8	122.5	3.75×10^{-4}	0.93	6.74×10^{-3}	0.142	134.0
**CdTe/CdS/TiO_2/Ni**	**0.01**	**36.2**	**8.7**	**2.48×10^{-3}**	**0.83**	**1.04×10^{-4}**	**0.43**	**44.9**
